# Primary health care centers, extent of challenges and demand for oral health care in Riyadh, Saudi Arabia

**DOI:** 10.1186/s12913-016-1876-6

**Published:** 2016-11-04

**Authors:** Abeer Al-Jaber, Omar B. Da’ar

**Affiliations:** 1 Quality & Accreditation Unit, Riyadh Public Health Administration, Ministry of Health, Riyadh, Saudi Arabia; 2 Department of Health Systems & Quality Management, College of Public Health & Health Informatics, King Saud Bin Abdulaziz University for Health Sciences, Riyadh, Saudi Arabia; 3 Adjunct Assistant Professor, Graduate School of Professional Studies, St. Mary’s University of Minnesota, Miineapolis, Minnesota USA

**Keywords:** Primary healthcare, Oral health care, Challenges, Demand, Cumulative dental visits, Ordinal regression, Saudi Arabia

## Abstract

**Background:**

As primary health care (PHC) centers in Saudi Arabia provide standardized preventive and curative oral health care (OHC) services, challenges remain. In addition, evidence of determinants of OHC seeking behavior is unknown. The aim of this study was to identify common challenges faced by patients seeking OHC in PHC centers and assess determinants of demand for OHC in Riyadh.

**Methods:**

After institutional approval and piloting, 320 adult patients were sampled at two large PHC centers in October 2015. Using a modified version of General Practice Assessment (GAPQ) and New York State Department of Health (NYSDOH) Questionnaires, information about cumulative number of visits to a dentist, patient profiles, provider characteristics, and challenges were collected. We used descriptive statistics to summarize data and employed ordinal regression for analyzing extent of effects of challenges and determinants of demand for OHC.

**Results:**

Oral health condition was reported to be good in 31 % of the patients, very good in 25 % of the patients, and fair in 20 % of the patients. More than half (53 %) of patients visited a dentist in the past 12 months once, 20 % twice, and 25 % at least three times. High cost of private clinic and unavailability of dentists were reported as the most common difficulties in seeing a dentist. Patients who were very satisfied with dental care and treatment plan, those with less than excellent oral health conditions and male patients had less number of cumulative visits to a dentist compared with patients with less satisfaction, patients with perceived excellent oral health, and female patients respectively.

**Conclusions:**

Our findings provide a strong evidence of challenges faced by patients and determinants of demand for OHC seeking behavior. The findings can inform policy maker not only in patient satisfaction of OHC, but also implications on facilities and health care systems. We conclude with recommendations for future research, especially on oral health preventive measures in PHC centers that correct inherent dental problems and other underlying challenges.

## Background

Globally, there have been great improvements in population health following the 1978 World Health Organization (WHO) *health for all* primary health care (PHC) strategy in Alma Ata. However, according to a 2002 World Health report, significant risks to population health in terms of burden of disability, diseases and deaths remain [1]. WHO Department of Non-communicable Disease Prevention and Health Promotion (NPH) developed a technical program based on the prevention of new diseases and strategy for health promotion. One of these programs is WHO Global Oral Health Program that collaborates with other programs of NPH and external partners to develop global policies promoting oral health and prevention of oral diseases [2].

These policies consider oral health care (OHC) as essential to the overall health and wellbeing by minimizing oral-facial diseases and other abnormalities [2]. In addition, OHC enhances life quality given that the craniofacial system represented by human faces allow people to enjoy life with a smile, eat, smell, or even cry [2]. Oral health has been linked to general health as evidenced by the relationship between periodontal diseases and diabetes mellitus [3]. Furthermore, there is evidence that a proper oral care and early detection of oral diseases not only reduce mortality rates and save patients’ lives [2], but also save costs [4].

Currently, oral health services in several countries are integral parts of change processes in general health to improve access, provide full coverage, increase efficiency, and improve the quality and safety of OHC [2]. Despite evidence of relationship between improved OHC and better access and effective utilization [5], many countries are still far from offering free oral health for all. Unfortunately, this is a reality nearly four decades after the adoption of WHO health for all strategy.

In line with international accords to provide standardized preventive and curative health services, Saudi Arabia in 1980 adopted WHO ‘health for all’ strategy and declared PHC as a cornerstone to achieve that goal [6]. As one of the most important strategies of Ministry of Health (MOH), the country has since adjusted her health care system according to Alma Ata declaration [7]. Yet, basic OHC as an essential component of PHC services still fall short of patient expectations in the country. Lack of appropriate geographic distribution of PHC centers often cause overcrowding in some centers and underutilization in others, causing a mismatch between PHC services and population needs. This imbalance poses a challenge to the health care system, especially mobilization of resources. Despite the free access to health care in Saudi Arabia, only a small percentage of adults go for routine checkups. Underutilization of OHC services might reflect underlying challenges in the provision of OHC, a possible reason why caries prevalence in primary and permanent teeth [8] and deepened periodontal pockets still remain high among the population [9].

There is an increasing focus on oral hygiene in Saudi Arabia however, because of growing demand for better oral health care, although the need varies from one group to another. Residents are increasingly seeking routine OHC checkups.

In general, dental clinics in Saudi Arabia provide preventive and curative services. About 2,408 dental clinics provide oral health care according to health statistic annual book. These providers are classified as clinics in PHC centers, dental clinics located within hospitals, and dental clinics in specialized centers (1, 2). There are also portable dental clinics that provide similar services (2).

While PHC centers play a central role in Saudi Arabia, they face numerous challenges in their strategies to provide OHC. In the wake of these challenges, the extent of the effect of demand and supply-related factors on OHC seeking behavior is unknown.

This paper therefore aimed to identify challenges faced by patients seeking OHC in PHC centers and assess determinants of demand for OHC services in Riyadh, Saudi Arabia.

## Methods

This is a cross-sectional study conducted at two selected PHC centers under Ministry of Health in Riyadh. In October 2015, we randomly sampled and administered 320 questionnaires to adult patients (≥18 years old) who were seeking OHC in PHC centers. The selected PHC centers are usually crowded because of lack of appropriate geographic distribution and proximity to the capital city. Thus, our sample was convenient. Besides, the selected centers enabled inclusion of some patients who are difficult to survey in their homes and workplaces because of cultural reasons.

The data were collected using a questionnaire developed based on General Practice Assessment Questionnaire (GAPQ) and New York State Department of Health (NYSDOH). We modified the questionnaire to fit local context. Once sampled, participants were invited to fill questionnaires in a waiting area with help of research assistants and a trained co-investigator. The questionnaires were translated into a local language and collected if completed within the waiting time. To ensure all participants properly completed questionnaires, follow-ups were made with help of a provider staff. Three hundred and five (305) questionnaires were returned, a response rate of 95 %. After excluding five incomplete ones, we effectively analyzed 300 questionnaires. The questionnaire contained socioeconomic and demographic profiles, OHC conditions, utilization and OHC-seeking behavior and challenges, as well as characteristics of facilities and providers. Data were collected from patients five days a week for a month.

The study was approved by both the Institutional Review Board (IRB) of MOH/King Fahd Medical City (KFMC) – Ref/15-313E and IRB –Ref/SP/094/15 of King Abdulaziz International Medical Research Center (KAIMRC).

The data were entered into MS Excel spreadsheet and transferred to STATA version 12 for analysis. We used descriptive statistics to summarize data and employed ordinal regression for estimating determinants of OHC seeking behavior. Patients reported one, two, and three or more visits to a dental facility. We used this measure of cumulative number of visits as our dependent variable. Since the dependent variable had more than two categories with meaningful sequential order values, ordinal regression was deemed necessary. The explanatory variables used were derived from patient profiles and provider characteristics consistent with behavioral model of health services use. These variables are descriptively summarized in the results section.

## Results

### Descriptive statistics

Table 1 summarizes demographic and socioeconomic profiles of participants. Of the 300 patients analyzed, 34 % were between 20 and 34 years, while 14 % were in the category of under 20 years age. About 47 % of the sampled patients had family size of more than six members, while 8 % had two persons in their households. Nearly one-fourth (24 %) of the respondents reported having a household income of between 2,000SR to 4,000SR. Another 24 % of participants reported having a household income of between 6,000SR to 8,000SR, while 12 % reported a household income of more than 10,000 SR. Females were 72 % of the participants. About 37 % of participants had college degree, while 33 % had high school level of education. About 71 % of participants indicated that they were healthy. Thirty-nine percent of the sampled patients had employment, while 22 % were homemakers. Nearly a fifth (18 %) were students, while 6 % of patients were retired.

**Table 1:** Patients’ demographic and socioeconomic profiles

Variable Definition		*N*	Percentage (%)
No. of times visited dentist in the past 12 months	One time	159	53.9 %
	Two times	60	20.3 %
	Three or more times	76	25.8 %
Age	Less than 20	41	13.7 %
	From 20–34	102	34.0 %
	From 35–44	78	26.0 %
	More than 45	79	26.3 %
Household income	2000–4000	72	24.0 %
	4001–6000	63	21.0 %
	6001–8000	73	24.3 %
	8001–10000	48	16.0 %
	Above 10001	36	12.0 %
Education	Less than High school	85	28.3 %
	High school	100	33.3 %
	College degree	110	36.7 %
Gender	Male	80	26.7 %
	Female	213	71.0 %
Family size	1	5	1.70 %
	2	24	8.0 %
	5-Mar	119	39.7 %
	More than 6	140	46.7 %
Working status	Employed	116	38.7 %
	Unemployed	35	11.7 %
	At school	55	18.3 %
	Looking after your family	66	22.0 %
	Retired	19	6.3 %
	Others	9	3.0 %

Table 2 shows frequency and reasons of dental visits. Nearly three-fifth (61 %) of patients visited dentist in less than one year, while 22 % did so within one to two years, and 14 % for more than two years. Moreover, about 53 % of the participants visited a dentist in the past 12 months once, 20 % twice, and 25 % at least three times. The reasons for visiting dentist were tooth or gum problems in (51 %), dentures (19 %), regular checkup (15 %), and braces (11 %).

**Table 2:** Patients’ frequency and reasons of dental visits

Variable Definition		*N*	Percentage (%)
The last time patients visited a dentist	Less than 1 year ago	184	61.3 %
	Between 1 and 2 years ago	65	21.7 %
	Over 2 years ago	41	13.7 %
	Never	7	2.3 %
Number of times visited a dentist in the past 12 months	1 time	159	53.0 %
	2 times	60	20.0 %
	3 or more times	76	25.3 %
Reason for visiting dentist last time	Regular check-up/cleaning	45	15.0 %
	Tooth or gum problem	152	50.7 %
	Dentures	57	19.0 %
	Braces	34	11.3 %

**Fig. 1:**
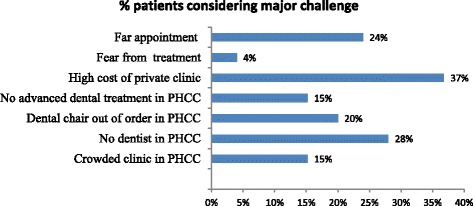
Major difficulties in seeking dental care

**Fig. 2:**
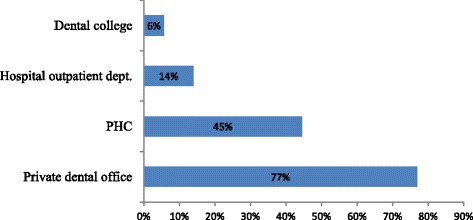
Where patients typically receive dental care

The major difficulties in seeking dental care summarized in Fig. 1. High cost of private dental clinics (37 %) was reported as the most common difficulty, followed by unavailability of a dentist in PHC centers (28 %). In terms of where patients have visited for care, 77 % sought care at private dental clinics while 45 % frequently visited PHC centers (see Fig. 2).

### Ordered logistic regression

This section presents the results of the estimations of ordered logistic regression model. Our analytical sample consists of those individuals who sought dental care in the past 12 months. The unit of analysis is the patient seeking OHC in PHC centers. Our reference patient category is a female who at least made a visit in the past year, aged 35 to 44, and has less than college education. The reference patient is also unemployed, comes from a household with a minimum of two members and household income of between 10,001 and 15,000. The reference patient further has good oral health condition, waited for treatment for less than a month, and is somewhat satisfied with dental care.

Ordinal regression revealed that male patients are less likely to have a higher cumulative number of visits to a dentist (*p* = 0.017) than female (Table 3).

**Table 3:** The effect of patient profiles and provider factors on OHC demand

		Estimate	Std. Error	Wald	Df.	Sig.	95 % Confidence Interval	
							Lower Bound	Upper Bound
Threshold	One visit last year	16.691	1.861	80.423	1	0	13.043	20.339
	2 visits last year	17.705	1.86	90.604	1	0	14.059	21.35
	Distance	−0.051	0.047	1.163	1	0.281	−0.143	0.041
	Family Size	0.005	0.09	0.004	1	0.953	−0.171	0.181
Age	<20	0.446	0.517	0.744	1	0.388	−0.567	1.459
	20–34	0.025	0.376	0.004	1	0.947	−0.713	0.762
	35–44	0.161	0.388	0.173	1	0.678	−0.599	0.921
	>45	0^a^	.	.	0	.	.	.
House income	2000–4000	−0.34	0.537	0.401	1	0.527	−1.392	0.712
	4001–6000	0.217	0.538	0.163	1	0.686	−0.838	1.272
	6001–8000	−0.005	0.506	0	1	0.992	−0.997	0.986
	8001–10000	−0.06	0.558	0.011	1	0.915	−1.154	1.034
	>10001	0^a^	.	.	0	.	.	.
Education	Less High school	0.47	0.44	1.143	1	0.285	−0.392	1.332
	High school	0.295	0.381	0.599	1	0.439	−0.452	1.042
	Collage degree	0^a^	.	.	0	.	.	.
Gender	Male	−0.92	0.387	5.658	1	0.017	−1.678	−0.162
	Female	0^a^	.	.	0	.	.	.
Transportation	Always	−0.69	0.576	1.433	1	0.231	−1.82	0.44
	Occasionally	−0.857	0.797	1.155	1	0.282	−2.419	0.706
	Sometimes	−0.405	0.531	0.582	1	0.446	−1.446	0.636
	Never	0^a^	.	.	0	.	.	.

Link function: Logit

^a^This parameter is set to zero because it is redundant

The results of the ordinal regression showed that patients who reported “excellent” oral health are more likely to have a higher cumulative number of visits to a dentist (*p* = 0.053) than patients who reported “Good” oral health (*p* = 0.075) – see Table 4. Patients who reported being very and somewhat satisfied with dental care and treatment plan are less likely to have a lower cumulative number of visits to a dentist (*p* = 0.006 and *p* = 0.000) relative to unsatisfied patients (Table 4). In addition, patients who reported being “indifferent” about their oral health treatment plan are less likely to have a lower cumulative number of visits to a dentist (*p* = 0.036). This result is, however of marginal statistical significance (Table 4).

**Table 4:** The effect of patient profiles and provider factors on OHC demand

		Estimate	Std. Error	Wald	Df.	Sig.	95 % Confidence Interval	
							Lower Bound	Upper Bound
Threshold	One visit last year	16.691	1.861	80.423	1	0	13.043	20.339
	2 visits last year	17.705	1.86	90.604	1	0	14.059	21.35
	Distance	−0.051	0.047	1.163	1	0.281	−0.143	0.041
	Family Size	0.005	0.09	0.004	1	0.953	−0.171	0.181
Oral health condition	Excellent	1.244	0.643	3.738	1	0.053	−0.017	2.504
	Very good	0.213	0.459	0.215	1	0.643	−0.686	1.112
	Good	−0.756	0.425	3.17	1	0.075	−1.588	0.076
	Fair	0.161	0.44	0.133	1	0.715	−0.701	1.023
	Poor	0^a^	.	.	0	.	.	.
Waiting time for Treatment	<1 month	0.629	0.46	1.866	1	0.172	−0.273	1.531
	1–3 months	0.359	0.454	0.624	1	0.43	−0.531	1.249
	>3 months	0^a^	.	.	0	.	.	.
Dental care satisfaction	Very satisfied	−2.17	0.793	7.48	1	0.006	−3.725	−0.615
	Somewhat satisfied	−2.06	0.591	12.159	1	0	−3.218	−0.902
	Neutral	−1.164	0.556	4.386	1	0.036	−2.252	−0.075
	Somewhat dissatisfied	−0.209	0.566	0.136	1	0.712	−1.317	0.9
	Very dissatisfied	0^a^	.	.	0	.	.	.
Average dentist satisfaction	Very good	−0.379	1.195	0.1	1	0.751	−2.72	1.963
	Good	−0.356	1.192	0.089	1	0.765	−2.692	1.979
	Satisfactory	−0.509	1.262	0.163	1	0.687	−2.982	1.964
	Poor	0.652	1.739	0.141	1	0.708	−2.757	4.062
	Very poor	0^a^	.	.	0	.	.	.

Link function: Logit

^a^This parameter is set to zero because it is redundant

Finally, the results indicated that patients who reported, ‘dental chair working properly’ are more likely to have a higher cumulative number of visits to a dentist (*p* = 0.030) – see Table 5. Surprisingly, there was no statistical evidence to suggest that satisfaction with dentists or providers, waiting time of treatment, and major challenges affected cumulative number of visits to a dentist.

**Table 5:** The effect of patient profiles and provider factors on OHC demand

		Estimate	Std. Error	Wald	Df.	Sig.	95 % Confidence Interval	
							Lower Bound	Upper Bound
Threshold	One visit last year	16.691	1.861	80.423	1	0	13.043	20.339
	2 visits last year	17.705	1.86	90.604	1	0	14.059	21.35
	Distance	−0.051	0.047	1.163	1	0.281	−0.143	0.041
	Family Size	0.005	0.09	0.004	1	0.953	−0.171	0.181
Major difficulties	Crowded =0	1.076	0.676	2.536	1	0.111	−0.248	2.4
	Crowded =1	0^a^	.	.	0	.	.	.
	No dentist = 0	−0.324	0.432	0.564	1	0.453	−1.17	0.522
	No dentist =1	0^a^	.	.	0	.	.	.
	Chairout =0	1.262	0.583	4.684	1	0.03	0.119	2.404
	Chairout =1	0^a^	.	.	0	.	.	.
	No Adv.tx = 0	−0.746	0.58	1.655	1	0.198	−1.883	0.391
	No Adv.tx = 1	0^a^	.	.	0	.	.	.
	Cost =0	0.006	0.379	0	1	0.987	−0.737	0.75
	Cost = 1	0^a^	.	.	0	.	.	.
	Fear =0	17.555	0	.	1	.	17.555	17.555
	Fear = 1	0^a^	.	.	0	.	.	.
	Far appoint = 0	−0.176	0.519	0.115	1	0.735	−1.193	0.841
	Far appoint =1	0^a^	.	.	0	.	.	.

Link function: Logit

^a^This parameter is set to zero because it is redundant

## Discussion

Our study examined the challenges faced by patients and determinants of demand for OHC services in PHC centers. Key findings include high cost of private clinic (36.8 %) and unavailability of dentists in PHC centers (28 %) as the main difficulties in the utilization of OHC services. The unavailability of a dentist in PHC centers perhaps explains why most of the sampled patients indicated visiting private dental clinic more frequently (77 %) than public PHC centers (45 %). Patients reported lack of advanced dental treatments in PHC centers given that these centers are known for primary dental treatment [10]. While these results may reveal unmet patients’ needs in PHC centers, the issue is common in many countries across the globe [5, 11–15].

Our study also revealed that patients who reported being very and somewhat satisfied with dental care and treatment plan have a fewer number of visits to a dentist (*p* = 0.006 and *p* = 0.000) relative to unsatisfied patients. This finding is consistent with previous studies, which revealed that less satisfaction is reported by people who visit dentists only for pain relief [16], while other studies showed patient satisfaction increased with more OHC utilization and dental visits [17, 18]. Further, our study revealed that male patients are less likely to have a higher cumulative number of visits to a dentist (*p* = 0.017) as compared to female, which is consistent with findings of previous studies that female patients visit dentist more frequently to utilize OHC services [19].

Finally, our results showed that patients with excellent oral health report more dental visits (*p* = 0.053) compared with those with poor oral health conditions. This result is intuitive and consistent with other studies, which report that more dental visits improve oral health [20–22].

While our study provides important data about OHC challenges in PHC centers, there are a number of limitations however. First, as is common with surveys, there might be recall biases relating to accessibility in terms of number of visits and challenges faced by patients when seeking OHC services. Additionally, although the two selected PHC centers helped inclusion of patients who are culturally difficult to access, our analysis is also limited insofar as the generalizability of the results is concerned. Contrary to a priori expectations, some key variables such as the main challenges faced by patients, satisfaction with dentists, and waiting time did not affect cumulative number of visits, a limitation that may point to estimation issues. These estimation limitations might be also due to some interactions of independent variables, thus biasing outcome.

## Conclusions

Our study identified challenges faced by patients seeking OHC in PHC centers and assessed determinants of demand for OHC services as measured by the cumulative number of visits. Main challenges faced by patients include high cost of private dental clinics, unavailability of dentists, unavailability of appointment slots, and crowded clinics. Contrary to a priori expectation, these challenges, satisfactions dentists and waiting time of treatment did not explain variations in the number of cumulative visits. Instead, satisfaction with dental care and treatment plans, conditions of oral health, and gender significantly explained variations in the cumulative number of visits to a dentist or facility. Patients expressing higher satisfaction with dental care and treatment plans were less likely to have a lower cumulative number of visits to a dentist than very unsatisfied patients. In addition, while male patients are less likely to have a higher cumulative number of visits to a dentist than females, patients with more visits are more likely to report excellent oral health conditions. Our study revealed that when key equipment such as dental chair work properly, patients make more visits to a dentist.

The findings of our study can inform management and public policymaking on understanding determinants of OHC seeking behavior, especially in countries where primary oral health services fall short of patient expectations. We therefore conclude with recommendations of a future research to investigate why some common challenges faced by PHC centers, satisfaction with dentists, or length of waiting time of treatment do not necessarily explain variations in cumulative visits to an OHC provider.

## Abbreviations

IRB: Institutional Review Board (IRB)
KAIMRC: King Abdul-Aziz International Medical Research Center
KFMC: King Fahd Medical City
MoH: Ministry of Health (MoH)
NPH: Non-communicable disease prevention and health promotion
OHC: Oral health care
PHC: Primary health care
WHO: World Health Organization
SR: Saudi Riyal.
